# Association of complete uncinate process removal on 2-year assessment of radiologic outcomes: subsidence and sagittal balance in patients receiving one-level anterior cervical discectomy and fusion

**DOI:** 10.1186/s12891-020-03443-7

**Published:** 2020-07-06

**Authors:** Sung Hyun Noh, Jeong Yoon Park, Sung Uk Kuh, Dong Kyu Chin, Keun Su Kim, Yong Eun Cho, Kyung Hyun Kim

**Affiliations:** 1 Department of Neurosurgery, National Health Insurance Service Ilsan Hospital, Goyang, South Korea; 2 Department of Neurosurgery, Yonsei University College of Medicine, Seoul, South Korea; 3 Department of Neurosurgery, Spine and Spinal Cord Institute, Gangnam Severance Hospital, Yonsei University College of Medicine, Seoul, South Korea

**Keywords:** Anterior cervical discectomy and fusion, Subsidence, Uncinate process resection, Instability

## Abstract

**Background:**

Many patients with cervical radiculopathy experience stenosis of the neural foramens due to cumulative osteophyte or uncovertebral joint hypertrophy. For cervical foraminal stenosis, complete uncinate process resection (UPR) is often conducted concurrently with anterior discectomy and fusion (ACDF). The aim of this study was to assess the clinical and radiological outcomes of ACDF with complete UPR versus ACDF without UPR.

**Methods:**

In total, 105 patients who performed one-level ACDF with a cage-and-plate construct between 2011 and 2015 were retrospectively reviewed. Among them, 37 patients had ACDF with complete UPR, and 68 patients had ACDF without UPR. Radiologic outcomes of disc height, C2–C7 lordosis, T1 slope, C2–C7 sagittal vertical axis (SVA), center of the sella turcica–C7 SVA (St-SVA), spino-cranial angle (SCA), and fusion rate were evaluated on plain X-ray at pre-operation, immediately post-operation, and at 2-year follow-up. For statistically matched pairs analysis, ACDF with UPR group (24 patients) and ACDF without UPR (24 patients) were compared.

**Results:**

All of the clinical parameters improved at the 2-year follow up (*P* < 0.0001). Improvement in visual analogue scale (VAS) scores for arm pain was significantly improved in the ACDF with complete UPR group immediately post-operation. All cervical sagittal parameters, including cervical lordosis, segmental angle, disc height, C2-C7 SVA, St-SVA, T1 slope, and SCA, except for preoperative St-SVA, SCA, and disc height of 2 years follow-up, were similar between the ACDF with complete UPR and ACDF without UPR groups. Differences in disc height, C2-C7 SVA, and SCA at 2-year follow up after preoperative examination, however, were statistically significant (*p* < 0.05). Subsidence occurred in 9 patients (ACDF with complete UPR: 8 cases [33%] versus ACDF without UPR: 1 cases [4%]; *p* < 0.05).

**Conclusions:**

Cervical sagittal alignment after ACDF with complete UPR is not significantly different from that achieved with ACDF without UPR. However, subsidence appears to occur more often after ACDF with complete UPR than after ACDF without UPR, although with little to no clinical impact. More precise and careful selection of patients is needed when deciding on additional complete UPR.

## Background

Anterior cervical discectomy and fusion (ACDF) aiming to improve the stability of the vertebra by decompression of neural elements and fusion is regarded as the gold-standard procedure for symptomatic cervical spondylosis in patients in whom non-operative care has failed [1]. Clinical and radiologic results after ACDF appear to be good [2]. Many patients with cervical radiculopathy also experience stenosis of the neural foramens because of cumulative osteophyte or uncovertebral joint hypertrophy. Although most anterior cervical discectomy and fusion procedures include cervical uncosectomy or uncoforaminotomy to decompress nerve roots in patients with cervical radiculopathy, Lee DH et al. reported that complete uncinate process resection (UPR) during ACDF improves pain in a patient’s arm more rapidly than conventional ACDF without UPR and provides similar fusion rates [3, 4]. Meanwhile, SH Lee et al. reported that complete UPR over 38% during ACDF increases the risk of subsidence during follow up [5].

At present, there is little evidence of whether this surgical technique provides good clinical and radiologic outcomes after complete unilateral or bilateral UPR, especially in regards to subsidence and cervical sagittal alignment. Accordingly, this study was undertaken to evaluate the association of complete UPR on subsidence and regional cervical sagittal balance by comparing the clinical and radiologic outcomes after ACDF with complete UPR versus ACDF without UPR.

## Methods

### Patient recruitment and inclusion criteria

**Fig. 1:**
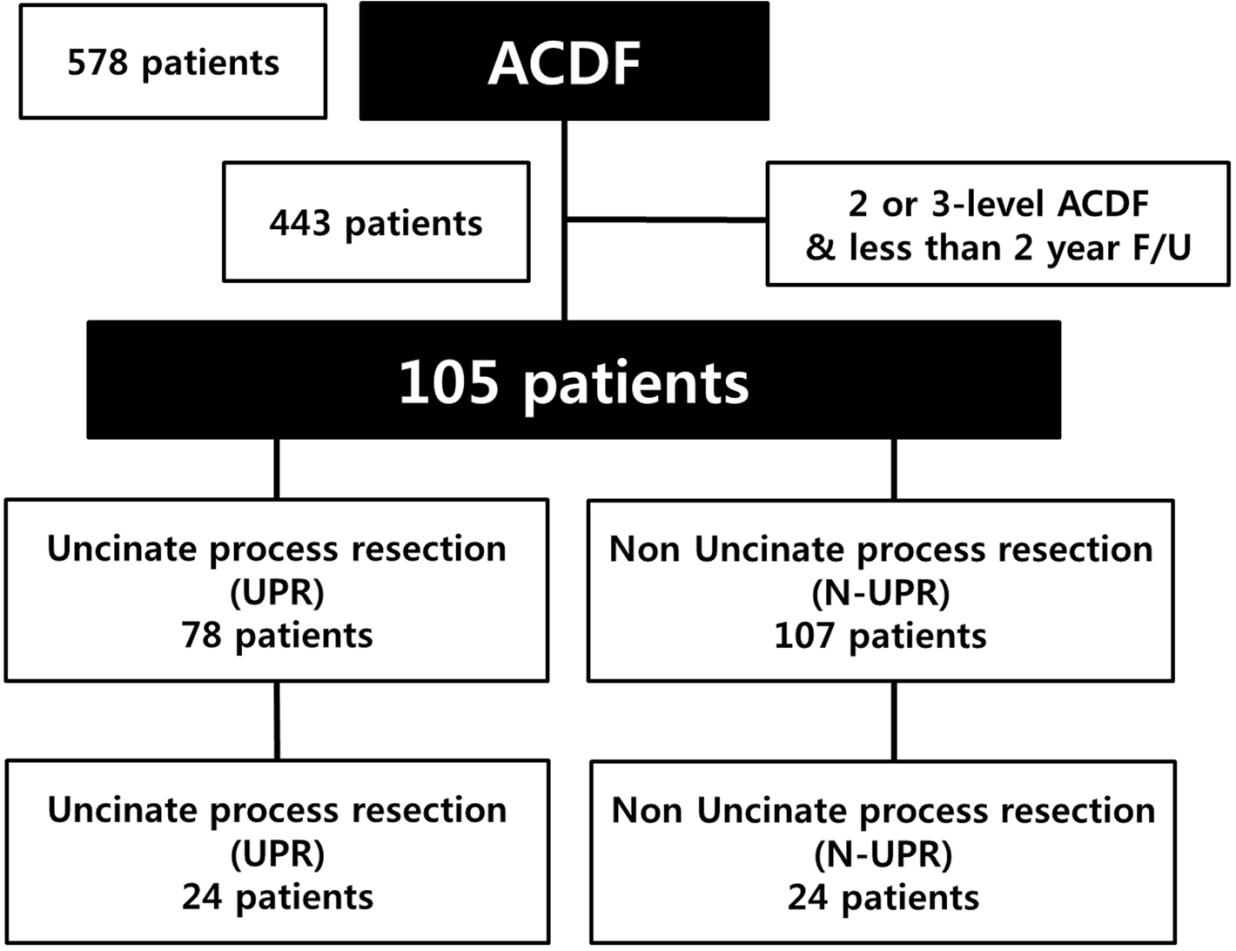
Flow chart of the patients in our study

**Fig. 2:**
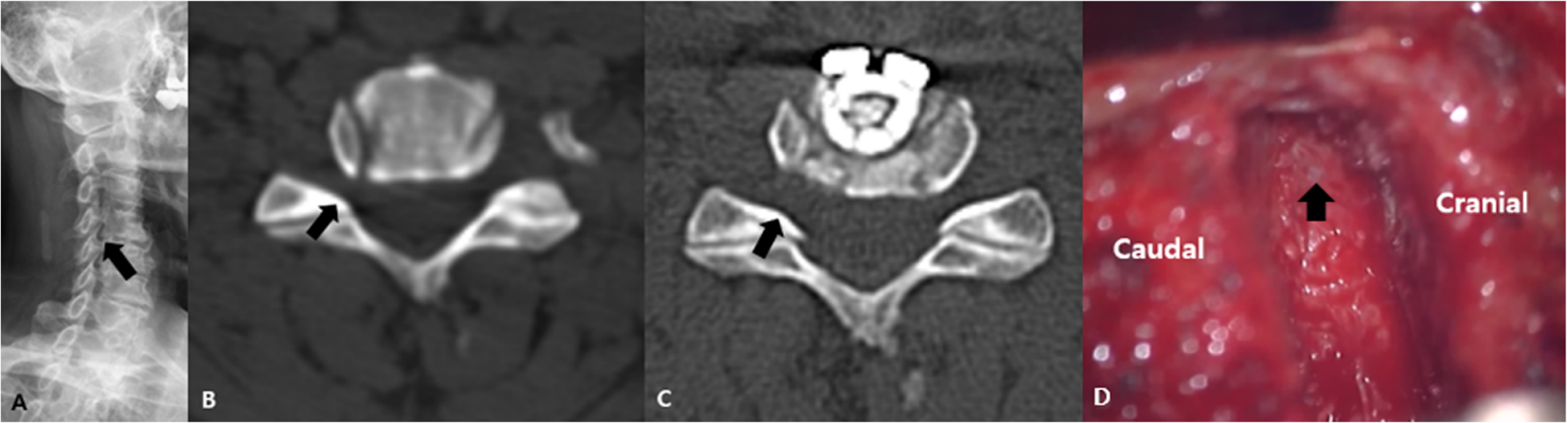
A: Cervical spine oblique radiographs at C4–5 (black arrow). B: Cervical spine CT (axial view) shows right foraminal stenosis (black arrow). C: ACDF with UPR was performed, and the right foramen was widened on post-operative CT (black arrow). D: The nerve root was decompressed by completely removing the uncinate process (black arrow)

Between January 2011 and December 2015, 578 patients who underwent ACDF for cervical spondylotic disease at our institution were collected. Among them, we excluded 473 patients whose follow-up period was less than 2 years or the surgery level was two levels or more. In this retrospective study, 105 consecutive patients with single-level cervical spondylotic disease who underwent primary ACDF with a cage-and-plate construct between January 2011 and December 2015 at the author’s institution were included (Fig. 1). This study was approved by the Institutional Review Board of our hospital. The uncinate process was randomly removed totally according to the technical preference of the single surgeon (Fig. 2). Thus, we defined ACDF with UPR as complete unilateral or bilateral removal of the uncinate process, while ACDF without UPR was defined as the conventional removal of only the anterior and posterior parts of the uncinate process or no removal of the uncinate process. This was confirmed with postoperative computed tomography scans. The patients were divided into two groups: 37 patients underwent ACDF with complete UPR and 68 patients were treated with ACDF without UPR. For statistically matched pairs analysis, ACDF with UPR group (24 patients) and ACDF without UPR (24 patients) were compared. The inclusion criteria included the following: 1) patients with symptoms of degenerative cervical disease; 2) patients who received primary ACDF with UPR at only one level; and 3) a follow-up period greater than 24 months. The exclusion criteria were as follows: 1) patients who had previous cervical spine surgery due to ossification of posterior longitudinal ligaments, fractures, tumors, etc.; 2) patients who underwent ACDF for more than two levels; and 3) a follow-up period less than 24 months.

### Surgical procedure

The patients were positioned under general anesthesia in the supine position. The surgical technique was chosen using a standard Smith–Robinson technique. After confirmation and exposure of the proper vertebral levels according to the compressive materials, a discectomy was performed, and a high-speed burr was applied to remove the anterior and posterior bony spurs and the endplate cartilage. The endplate cartilage was eliminated with a curette carefully to preserve the bony endplate as much as possible to prevent cage subsidence. Discs, endplate cartilaginous, and other compressive materials were subducted to achieve appropriate dural and neural decompression. Using an osteotome, a high-speed electric drill, and a Kerrison punch, the nerve roots were decompressed by completely removing the uncinate process. If the patient had unilateral symptoms and if radiologic results were consistent, we performed removal of the uncinate process unilaterally. We used a plate (Atlantis; Medtronic, Minneapolis, MN, USA) and allograft cage (Cornerstone®-SR; Medtronic, Minneapolis, MN, USA) with local autologous bone. We did not use autologous iliac bone or growth factors, such as demineralized bone matrix and recombinant bone morphogenetic proteins (rhBMP), as graft material. The proper size for the allobone cage was decided by both preoperative evaluation and intraoperative formatting using a trial cage. The cage was placed into the disc space as described above. Fixed type screw was utilized to fix the anterior cervical spine plate. If there was no complication during operation, all patients were able to sit upright and walk with a neck collar on the first day after surgery. The patients wore a cervical collar for 1 month after surgery. Clinical and radiographic results were obtained by an independent observer for 5 days post-operatively. In the outpatient clinic, patients were continuously followed up post-operation.

### Clinical outcome assessment

Intraoperative blood loss, operative time, days of hospitalization, and clinical outcomes were evaluated using the neck disability index (NDI), neck visual analog scale (VAS), and arm-VAS preoperatively, immediately after surgery, and at 2-year follow up. During the last follow up, the patient was assessed according to Odom’s criteria, from poor to excellent [6].

### Radiological evaluation

**Fig. 3:**
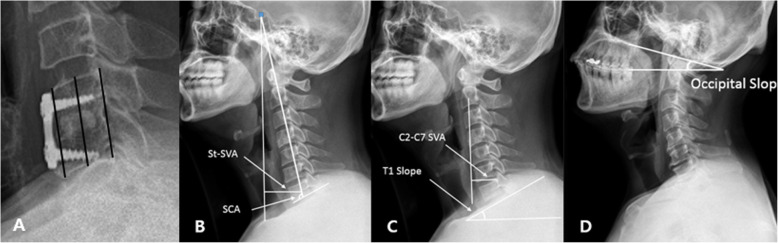
A: Subsidence measurements were performed from the anterior, middle, and posterior portions of the vertebral bodies of interest. Subsidence was described as a greater than 3 mm decrease in height of the operative segment between images produced immediately after the operation and those acquired at 2 years follow up. B: The SCA was defined as the angle between the C7 line and the line joining the center of the sellar turcica and the center of the inferior endplate of the C7 body. The center of the St-SVA was defined as the distance between a plumb line from the center of the sellar turcica and the center of the C7 body. C: The C2–C7 SVA was decided as the length from the posterosuperior corner of C7 and the vertical line from the center of the C2 body. The T1 slope was defined as the angle between the upper endplate of T1 and the horizontal line. D: O-s is the angle between the McGregor line and the horizontal line

Preoperative radiologic examination evaluated plain radiographs, computed tomography scans, and magnetic resonance imaging. Plain radiological examinations of the cervical spine were also conducted immediately after surgery and at 2-year follow up for all patients. Cervical alignment was evaluated using the Cobb angle of C2–C7, working the process described by Borden [7]: this angle was made by the lines along the inferior endplate of C2 to the inferior endplate of C7 in the neutral position. Subsidence was decided by measuring the distance from the upper endplate of the upper vertebral body to the lower endplate of the lower vertebral body at the level of the operation. The segmental angle was calculated using the Cobb angle of the adjacent vertebrae in the intervertebral disc involved. The total intervertebral height was decided as the length from the upper endplate of the cephalad vertebrae to the inferior endplate of the caudal vertebrae of the fused segment, which was quantified as the mean value of the height of the anterior and posterior borders [8]. Subsidence was described as a decline in the height of the operative segment greater than 3 mm between immediate images after the operation and those acquired at the last follow up (Fig. 3A). Spino-cranial angle (SCA) was defined as the angle between the C7 line and the line joining the center of the sella turcica and the center of the inferior endplate of the C7 body. The center of the sella turcica – C7 sagittal vertical axis (St-SVA) was defined as the distance between a plumb line hung from the center of the sella turcica and the center of the C7 body (Fig. 3B). The C2–C7 sagittal vertical axis (SVA) was decided as the length from the postero-superior corner of C7 and the vertical line from the center of the C2 body. The T1 slope was defined as the angle between the upper endplate of T1 and the horizontal line (Fig. 3C). Because keeping horizontal gaze is the most important function of the cervical vertebrae, patients maintained a horizontal gaze position during radiologic examination. Occipital slope (O-s) is a postural variable reflecting the position of the skull, and it can reflect the degree of horizontal gaze. O-s represents the angle between the McGregor line and horizontal line (Fig. 3D). We decided the maximum difference in the O-s values at each examination as 2 degrees. Radiological fusion was decided to have occurred when there was ≤2° movement on flexion–extension and/or ≤ 2 mm of movement of the interspinous distance on flexion–extension across the fusion segment [9].

### Statistical analysis

The findings are presented as mean values ± standard deviations (SD) or counts, as indicated. The independent t-test and chi-squared test results were used to compare both groups. By checking the normality of continuous data through Kolmogorov-Smirnov test, if the normality assumption is satisfied, the data are expressed as mean ± SD, and an independent two sample t-test is performed, and if the normality assumption is not satisfied, median (Q1-Q3), and Mann-Whitney U test was performed. The binary multiple logistic regression test was used to determine the influencing radiologic factors of subsidence as dependent variable. Gender, age, BMD, BMI, smoking, DM, operation level, resection side, and whether to remove uncinate as independent variables were adjusted and radiologic parameters were analyzed by binary multiple logistic regression. All *P* values < 0.05 were considered to indicate statistical significance. All statistical analyses were performed using SPSS (version 23.0, SPSS, Chicago, IL, USA).

## Results

### Patient demographics (Table 1)

In total, 105 patients underwent ACDF at the authors’ institution. Detailed demographics of 48 out of 105 patients were shown in Table 1. In the matched pair analysis, there was no statistically significant factor in the demographic between the two groups. The total of 105 patients’ ages ranged from 46 to 77 years (average age, 57.9 ± 11.83 years old). The patients were followed for an average of 37.7 ± 10.5 months. The operation level was primarily the C5/6 level (60 cases, 57%), followed by the C4/5 level (23 cases, 22%).

**Table 1:** Patient demographics

	ACDF without UPR (***n*** = 24)	ACDF with complete UPR (n = 24)	***p***-value
**Sex**			
**Female**	**15**	**16**	
**Male**	**9**	**8**	**0.763**
**Mean age (years)**	**47.9 ± 9.78**	**49.1 ± 9.67**	**0.669**
**BMD (g/cm**^**2**^**)** **T-score**	**−0.66 ± 1.21**	**−0.78 ± 0.77**	**0.681**
**BMI (kg/m**^**2**^**)**	**23.5 ± 2.47**	**23.5 ± 2.02**	**0.984**
**DM**	**5**	**6**	**0.731**
**Smoking**	**9**	**6**	**0.351**
**Operation level**			
**C2/3**	**0**	**0**	
**C3/4**	**6**	**5**	
**C4/5**	**15**	**15**	**0.999**
**C5/6**	**3**	**4**	
**C6/7**	**0**	**0**	
**Resection side**			
**Unilateral**		**20**	
**Bilateral**		**4**	

BMD; bone mineral density, BMI; body mass index, DM, diabetes mellitus;

ACDF; anterior cervical discectomy and fusion

UPR; uncinate process removal

*p < 0.05 comparing ACDF without UPR and ACDF with complete UPR

### Comparison of intraoperative blood loss, operative time, days of hospitalization, and clinical parameters (Table 2)

Intraoperative blood loss, operative time, days of hospitalization, Arm-VAS, Neck-VAS, NDI, and Odom’s criteria of the two groups are shown in Table 2. All of the clinical parameters improved at 2-year follow up (*P* < 0.0001). Regarding Odom’s criteria, most of the surgical results were excellent and good in both groups. Also, there was no complication in either group. There was no statistically significant clinical outcome between the ACDF with UPR and ACDF without UPR groups except for postoperative Arm-VAS.

**Table 2:** Comparison of intraoperative blood loss, operative time, days of hospitalization, and clinical parameters

	ACDF without UPR (n = 24) Median (Q1-Q3),(min-max) N(%)	ACDF with complete UPR (n = 24) Median (Q1-Q3),(min-max) N(%)	p-value
**Intraoperative blood loss (ml)**	**60.00(52.50–80.00),(50.00–100.00)**	**77.50(57.50–90.00),(50.00–140.00)**	**0.175**
**Operation time (min)**	**100.00(90.00–120.00),(90.00–150.00)**	**120.00(100.00–130.00),(90.00–155.00)**	**0.086**
**Duration of hospitalization (day)**	**6.00(6.00–7.00),(5.00–9.00)**	**6.00(6.00–7.00),(5.00–9.00)**	**0.866**
**Arm VAS**			
**Preoperation**	**9.00(8.00–9.00), (7.00–9.00)**	**8.50(8.00–9.00), (7.00–9.00)**	**0.116**
**Postoperation**	**4.00(3.00–5.00), (2.00–6.00)**	**3.00(2.00–3.50), (2.00–5.00)**	0.003*
**2 years follow-up**	**2.00(1.00–2.00), (1.00–3.00)**	**2.00(1.00–2.00),(1.00–3.00)**	**0.711**
**Neck VAS**			
**Preoperation**	**9.00(8.00–9.00), (7.00–9.00)**	**9.00(8.00–9.00), (7.00–9.00)**	**0.817**
**Postoperation**	**2.00(1.00–5.00), (1.00–5.00)**	**2.00(2.00–3.50), (2.00–5.00)**	**0.657**
**2 years follow-up**	**1.00(1.00–2.00), (1.00–3.00)**	**1.00(1.00–1.00), (1.00–2.00)**	**0.281**
**NDI**			
**Preoperation**	**38.00(37.00–41.50), (35.00–44.00)**	**40.50(37.50–42.00), (35.00–44.00)**	**0.464**
**Postoperation**	**24.00(21.00–25.00), (15.00–29.00)**	**22.00(19.00–25.00),(15.00–27.00)**	**0.514**
**2 years follow-up**	**14.00(13.50–16.50), (11.00–19.00)**	**13.50(11.00–15.00),(11.00–17.00)**	**0.069**
**Odom’s criteria**			
**Excellent**	**9**	**9**	**0.999**
**Good**	**15**	**14**	
**Fair**	**0**	**1**	
**Poor**	**0**	**0**	

VAS; Visual analog scale, NDI; Neck Disability Index

*p < 0.05 comparing ACDF without UPR and ACDF with UPR

### Comparison of radiologic parameters (Table 3)

**Fig. 4:**
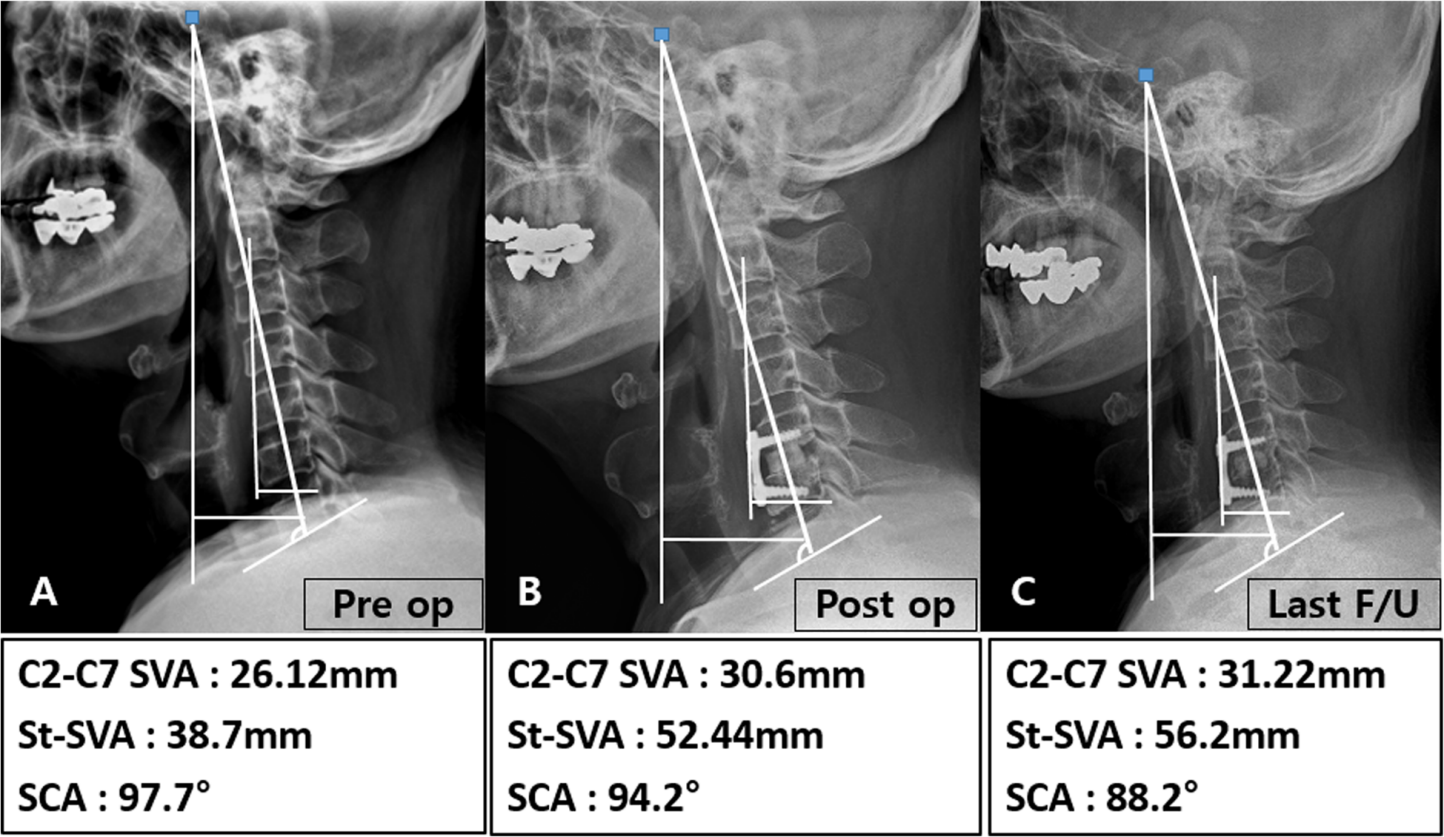
A case from the ACDF with complete UPR group. The patient underwent an ACDF operation of C5/6 with complete UPR. In this patient, C2–C7 SVA and St-SVA increased with time, but SCA decreased with time

**Fig. 5:**
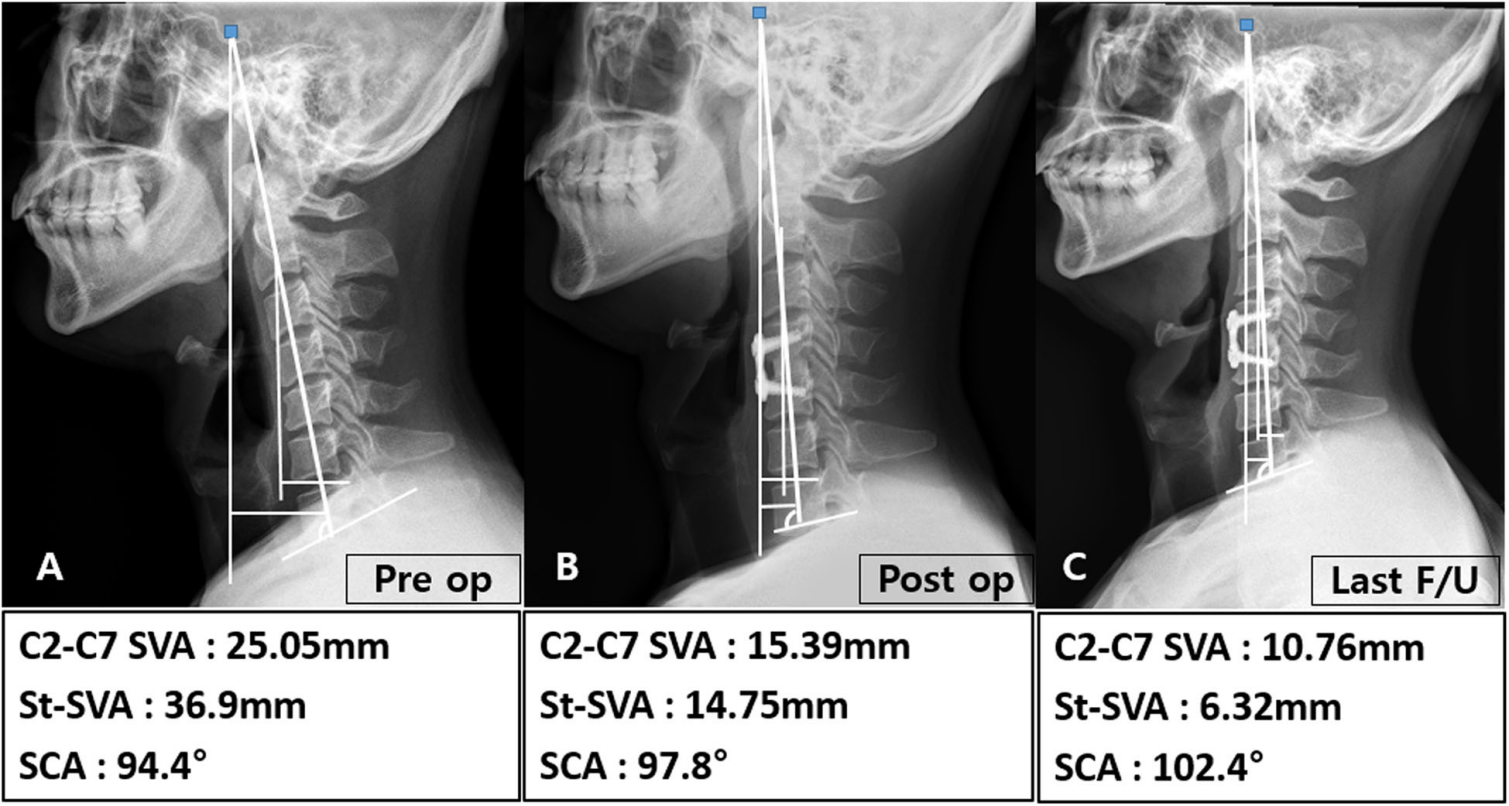
A case from the ACDF without UPR group. The patient underwent an ACDF operation of C4/5 without UPR. In this patient, C2–C7 SVA and St-SVA decreased with time, but SCA increased with time

Cervical lordosis, segmental angle, disc height, C2-C7 SVA, St-SVA, T1 slope, SCA, incidence of subsidence, and fusion rate of the two groups are shown in Table 3. All cervical sagittal parameters, including cervical lordosis, segmental angle, disc height, C2-C7 SVA, St-SVA, T1 slope, and SCA, except for preoperative St-SVA, SCA, and disc height of 2 years follow-up, were similar between the ACDF with complete UPR and ACDF without UPR groups. Differences in disc height, C2-C7 SVA, and SCA at 2-year follow up after preoperative examination, however, were statistically significant (*p* < 0.05). Subsidence occurred in 9 patients (ACDF with complete UPR: 8 cases [33%] versus ACDF without UPR: 1 cases [4%]; p < 0.05). Radiological images for representative patients in each group are displayed in Figs. 4 and 5. There was no statistical significance because there were only a few cases of removal of uncinate on both sides. However, subsidence occurred more frequently in cases of removal of both sides than in cases of removing only one side.

**Table 3:** Comparison of radiologic parameters

	ACDF without UPR (n = 24) Mean ± SD, N(%)	ACDF with complete UPR (***n*** = 24) Mean ± SD, N(%)	p-value
**C2–C7 lordosis (**°**)**			
**Preoperation**	**15.50(9.45–17.60), (3.90–20.80)**	**14.10(5.45–19.55), (3.90–26.40)**	**0.781**
**Postoperation**	**15.80(10.25–17.90), (7.70–27.90)**	**17.25(8.50–19.10), (3.00–27.30)**	**0.772**
**2 years follow-up**	**15.95(13.45–24.15), (10.20–28.50)**	**14.65(11.00–29.70), (1.10–45.50)**	**0.877**
**2 years follow-up - Preoperation**	**3.80(0.70–8.50),(− 5.70–17.00)**	**5.20(− 3.60–15.30),(− 12.20–30.00)**	**0.984**
**Segmental angle (**°**)**			
**Preoperation**	**5.45(4.60–5.95), (1.80–7.10)**	**4.95(4.00–5.50), (1.30–7.10)**	**0.215**
**Postoperation**	**5.85(2.90–7.45), (0.50–14.40)**	**5.70(3.45–7.55), (1.00–14.40)**	**0.918**
**2 years follow-up**	**5.90(5.10–7.25), (1.20–9.60)**	**5.20(3.75–6.10), (0.90–10.00)**	**0.207**
**2 years follow-up - Preoperation**	**0.70(− 0.75–2.90), (− 4.80–5.30)**	**0.50(− 1.60–2.30), (− 4.80–6.90)**	**0.643**
**Disc height (mm)**			
**Preoperation**	**5.60(5.15–6.18), (4.23–7.90)**	**5.96(5.58–6.26), (5.18–6.97)**	**0.173**
**Postoperation**	**7.16(6.46–7.90), (5.84–8.91)**	**7.53(7.27–7.84), (6.52–8.91)**	**0.117**
**2 years follow-up**	**6.22(5.41–6.58), (4.82–13.12)**	**5.19(5.15–5.55), (5.01–5.82)**	< 0.001*
**2 years follow-up – Preoperation**	**0.08(− 0.52–2.02), (− 1.42–6.95)**	**− 0.44(− 1.15--0.19), (− 1.78–0.45)**	0.007*
**C2–C7 SVA (mm)**			
**Preoperation** **Postoperation**	**20.05(15.39–26.31), (12.46–30.53)** **19.33(13.92–26.13), (9.77–29.04)**	**17.06(15.15–24.72), (6.97–28.53)** **18.58(13.25–29.88), (6.72–39.25)**	**0.261** **0.877**
**2 years follow-up**	**15.78(12.36–21.51), (10.62–30.84)**	**17.28(11.18–29.57), (4.42–41.93)**	**0.703**
**2 years follow-up - Preoperation**	**−3.98(− 5.80--2.84), (− 9.62–7.01)**	**− 0.26(− 4.15–5.88), (− 5.32–15.96)**	0.005*
**St-SVA (mm)**			
**Preoperation**	**30.77(24.05–35.06), (15.71–42.98)**	**25.68(20.72–29.36), (13.25–55.43)**	0.018*
**Postoperation**	**27.65(17.49–28.63), (10.59–52.27)**	**28.94(17.12–30.42), (4.40–61.23)**	**0.414**
**2 years follow-up**	**24.53(11.82–32.77), (9.37–48.84)**	**28.56(11.53–41.36), (4.74–77.58)**	**0.496**
**2 years follow-up - Preoperation**	**−1.30(− 3.00–1.25), (− 14.90–2.90)**	**0.50(− 0.70–2.75), (− 15.40–7.10)**	**0.066**
**T1 slope (**°**)**			
**Preoperation**	**25.15(20.25–27.90), (12.00–31.60)**	**24.10(22.00–25.90), (11.90–44.00)**	**0.687**
**Postoperation**	**24.85(17.10–28.10), (13.60–32.80)**	**25.40(20.50–27.55), (14.80–32.50)**	**0.599**
**Last follow-up**	**23.50(17.60–27.00), (12.00–33.20)**	**25.80(20.75–28.00), (15.40–49.20)**	**0.327**
**Last follow-up - Preoperation**	**−1.30(− 3.00–1.25), (− 14.90–2.90)**	**0.50(− 0.70–2.75), (− 15.40–7.10)**	**0.066**
**SCA (**°**)**			
**Preoperation**	**104.65(101.20–108.65), (89.90–115.90)**	**111.05(107.85–114.70), (101.20–120.00)**	< 0.001*
**Postoperation**	**104.75(100.90–108.45), (94.60–117.00)**	**105.90(103.65–111.45), (95.50–113.60)**	**0.397**
**2 years follow-up**	**105.80(100.60–111.60), (92.80–115.50)**	**105.80(99.10–107.30), (87.30–121.40)**	**0.634**
**2 years follow-up - Preoperation**	**3.65(−4.50–8.35), (− 13.30–10.70)**	**−8.15(− 15.15–2.70), (− 20.10–9.80)**	0.004*
**Subsidence**	**1 (4%)**	**8 (33%)**	0.023*
**Fusion**	**22 (92%)**	**22 (92%)**	**0.999**

SVA; sagittal vertical axis, St-SVA; sellar turcica–sagittal vertical axis,

SCA; spinocranial angle

* Statistically significant

### Binary multiple logistic regression of the five measurements as significant parameters on subsidence (Table 4)

Radiologic factors that may potentially associate with subsidence were analyzed using binary multiple logistic regression test. The results are shown in Table 4. As an association factor of subsidence, preoperative SCA values were significant (*P* < 0.05). In opposition to our hypothesis, complete UPR was not a significant factor affecting subsidence.

**Table 4:** Binary multiple analysis of the five measurements as significant parameters on subsidence

Factor	Odds Ratio	95% CI	p-value
**Preoperative C2–C7 SVA**	**1.034**	**0.896–1.192**	**0.651**
**Preoperative St-SVA**	**0.946**	**0.863–1.037**	**0.238**
**Preoperative SCA**	**1.237**	**1.074–1.425**	0.003*
**Preoperative CL**	**0.950**	**0.817–1.104**	**0.503**
**Preoperative T1-slope**	**0.998**	**0.870–1.145**	**0.978**

SVA; sagittal vertical axis, St-SVA; sellar turcica–sagittal vertical axis,

SCA; spinocranial angle, CL; cervical lordosis, CI: confidence interval

* Statistically significant

## Discussion

ACDF is the treatment of choice for symptomatic cervical spondylosis in patients when conservative treatments, such as medication or physiotherapy, have failed [10]. Patients with arm pain with neural foramen stenosis due to osteophytes or hypertrophy of the uncovertebral joint should be treated with ACDF, as well as UPR. ACDF with complete UPR is known to improve pain in the arm better and faster [11]. However, inadequate removal of the uncinate process has been reported to contribute to poor outcomes in cervical spondylosis cases [12]. In our study, the ACDF with UPR group had better arm pain in the immediate post-operation period than the ACDF without UPR group.

As the uncinate process is an important structure to maintaining the stability of adjacent vertebral bodies in the spinal axis, we investigated whether sagittal alignment or subsidence is affected by removing the uncinate process. Subsidence occurs as a natural process during the course of an interbody fusion procedure and is described as settlement of a body with a higher elasticity modulus (e.g., graft, cage, spacer) into a body with lower elasticity modulus (e.g., vertebral body), leading to a change in spine structure [13]. However, upon excessive subsidence, interbody spaces are narrowed and kyphosis of the spine occurs. This introduces instability of the screw-plate and screw-bone (e.g., pull-out, change of angulation, breakage of the instrumentation) [13]. To the best of our knowledge, end-plate preparation, type of cage and size, multilevel fusion, recombinant human bone morphogenetic protein-2 (rhBMP-2), process of instrumentation, and bone quality are significant factors of subsidence [14]. In our study, when the ACDF with complete UPR and ACDF without UPR were compared under the same conditions, subsidence was significantly higher when complete UPR was performed after 3 years on average. Considering these reasons, it would seem that end-plate preparations would be performed more in the process of UPR in the ACDF with UPR group. However, between the ACDF with UPR and ACDF without UPR groups, clinical results except postoperative Arm-VAS were not significantly different. This is because the foramen is widened due to the UPR, such that, even if subsidence occurs, radiculopathy due to pressing of the root does not occur. Overall, in the case of one-level ACDF, it is difficult to find a significant adverse effect of subsidence. However, caution against subsidence is needed, and a large-scale and long-term follow-up study of multiple-level ACDF with UPR is necessary.

Sagittal balance has been suggested for cervical spine treatment. T1 slope determines the sagittal balance of the cervical spine, and this parameter is related with C2–C7 angle [15]. Previous studies have reported that C2-C7 lordosis is closely related to the other cervical and thoracic parameters (cervical lordosis, thoracic kyphosis) [16]. Cervical sagittal imbalance influences the health-related quality of life (HRQOL) of patients [17]. St-SVA and C2–C7 SVA are closely associated with the clinical results of neck pain and HRQOL [18]. The study by Tang et al. suggested that increasing cervical SVA is a cause for clinical concern of cervical malalignment as reflected by poor HRQOL scores [19]. In our study, C2-C7 lordosis, segmental angle, disc height, C2-C7 SVA, St-SVA, T1 slope, and SCA were not different between ACDF with UPR and ACDF without UPR group, although the differences significant in disc height, C2-C7 SVA, and SVA at last follow-up and preoperatively were statistically between the two surgery groups (*p* < 0.05). Accordingly, there were no differences in clinical outcomes between the two groups.

Global cervical spine lordosis was not influenced by single-level ACDF [20]. This is the natural mechanism of the human body, which keeps the head on a neutral axis in the optimal horizontal plane for the visiovestibular system and re-establishes sagittal balance [20]. In our study, single-level ACDF with UPR did not affect sagittal balance, although parameters of C2-C7 lordosis, segmental angle, disc height, C2-C7 SVA, St-SVA, and SVA were worse. Thus, long-term follow up and a large scale study of multiple-level ACDF with UPR or ACDF in kyphotic cervical spine are necessary. Technically, UPR usually proceeds from the inside to the outside. This technique needs to be performed carefully because of the possibility of injury to the nerve roots and vertebral arteries. It is recommended to use a punch rather than a drill when removing the lateral portion of the uncinated process.

### Limitations of this study

Our study had a few limitations. The matched pair number of patients who underwent removal of the uncinate process was small. Also, cases with a bilaterally UPR were rare. And, because our study did not have a randomized controlled design, we could not completely control the possibility of selection bias. Additionally, because our study size was small, we were limited in our ability to make comparisons between the groups for several factors known to affect prognosis. Failure to indicate the extent to which the uncinate process was removed as an objective indicator was also a limitation. However, the results of this study suggest that when performing ACDF with complete UPR, the risk of subsidence should be considered. Prospective studies will be conducted using well-guided evidence-based protocols with adequate controls.

## Conclusion

Cervical sagittal alignment after ACDF with complete UPR is not significantly different from that achieved with ACDF without UPR. However, subsidence appears to occur more often after ACDF with complete UPR than after ACDF without UPR, although with little to no clinical impact. More precise and careful selection of patients is needed when deciding on additional complete UPR.

## Data Availability

The datasets used and analyzed during the current study are available from the corresponding author on reasonable request.

## Abbreviations

ACDF: Anterior cervical discectomy and fusion
UPR: Uncinate process resection
VAS: Visual analog scale
NDI: Neck disability index
BMD: Bone mineral density
BMI: Body mass index
DM: Diabetes mellitus
SVA: Sagittal vertical axis
St-SVA: Sellar turcica- Sagittal vertical axis
SCA: Spinocranial angle
CT: Computed tomography
MRI: Magnetic resonance imaging
O-s: Occipital slope
RhBMP-2: Recombinant human bone morphogenetic protein-2
HRQOL: Health-related quality of life
